# Supplementary material for: Factors associated with SARS-CoV-2 infection among people living with HIV: Data from the Balearic cohort (EVHIA)
Source: PLoS One. 2024 Aug 7;19(8):e0308568. doi: 10.1371/journal.pone.0308568 (PMC11305541; doi:10.1371/journal.pone.0308568)
Supplement: Attachment — Submitted filename: Comments.docx [file pone.0308568.s001.docx]

There is controversy regarding the effect of COVID-19 infection on people living with HIV, so the impact of SARS-CoV-2 infection among people living with HIV (PLWH) has been a matter of research. In this study the authors calculated COVID-19 incidence and related factors among PLWH, also they evaluated vaccine coverage and related factors among PLWH. The results showed that the incidence of COVID-19 among PLWH was 153 cases per 1000 person-years which was more than the general population. Also, they showed that Men who have sex with men (MSM) were associated with an increased risk of SARS-CoV-2 infection, whereas African origin, tobacco use, and complete or booster vaccination coverage were negatively related to SARS-CoV-2 infection among PLWH. I think this is a valuable study, however, I have some minor comments:

1.	The method section stated that the paper is a descriptive retrospective study, however, I think your study is analytic.

2.	In Table 3 aHZR should be replaced by aHR.

3.	in case of citing more than one reference in the text, please insert all citations in Parentheses for example (5, 6) no (5)(6).

4.	In Table 1, the authors reported column percentages, however, I think row percentages can be more informative.

5.	The authors covered two research questions of COVID-19 incidence and vaccine coverage among PLHIV. I think the authors presented a lot of information in the manuscript; I suggest you present these two research questions in different manuscripts.
